# Accuracy assessment of Tri-plane B-mode ultrasound for non-invasive 3D kinematic analysis of knee joints

**DOI:** 10.1186/1475-925X-13-122

**Published:** 2014-08-26

**Authors:** Md Abdullah Masum, Mark Pickering, Andrew Lambert, Jennie Scarvell, Paul Smith

**Affiliations:** School of Engineering & IT, UNSW Canberra, Northcott Drive, Campbell, Canberra, Australia; Faculty of Health, University of Canberra, University Drive, Bruce, Canberra, Australia; Medical School, The Australian National University, Linnaeus Way, Canberra, Australia

**Keywords:** Motion analysis, Ultrasound, Image registration, Knee joint diagnostics

## Abstract

**Background:**

Currently the clinical standard for measuring the motion of the bones in knee joints with sufficient precision involves implanting tantalum beads into the bones. These beads appear as high intensity features in radiographs and can be used for precise kinematic measurements. This procedure imposes a strong coupling between accuracy and invasiveness. In this paper, a tri-plane B-mode ultrasound (US) based non-invasive approach is proposed for use in kinematic analysis of knee joints in 3D space.

**Methods:**

The 3D analysis is performed using image processing procedures on the 2D US slices. The novelty of the proposed procedure and its applicability to the unconstrained 3D kinematic analysis of knee joints is outlined. An error analysis for establishing the method’s feasibility is included for different artificial compositions of a knee joint phantom. Some *in-vivo* and *in-vitro* scans are presented to demonstrate that US scans reveal enough anatomical details, which further supports the experimental setup used using knee bone phantoms.

**Results:**

The error between the displacements measured by the registration of the US image slices and the true displacements of the respective slices measured using the precision mechanical stages on the experimental apparatus is evaluated for translation and rotation in two simulated environments. The mean and standard deviation of errors are shown in tabular form. This method provides an average measurement precision of less than 0.1 mm and 0.1 degrees, respectively.

**Conclusion:**

In this paper, we have presented a novel non-invasive approach to measuring the motion of the bones in a knee using tri-plane B-mode ultrasound and image registration. In our study, the image registration method determines the position of bony landmarks relative to a B-mode ultrasound sensor array with sub-pixel accuracy. The advantages of our proposed system over previous techniques are that it is non-invasive, does not require the use of ionizing radiation and can be used conveniently if miniaturized.

## Background

Kinematic analysis allows the motion of individual bones in the knee joint to be measured [1, 2]. These motion measurements provide significant insights into normal and abnormal joint trajectories which can lead to improved artificial joint component design. They also allow improved diagnosis for ligament injuries and facilitate accurate therapeutic strategies to be formulated. Knee joint kinematics is complex in a sense that the natural motions of flexion, extension and internal, external rotation are coupled [3, 4]. During flexion, the two main bones in a knee joint, do not bend like a door hinge, instead they rotate about several specific axes. This type of motion makes it crucial that the joint kinematics be evaluated in 3D space.

Currently, the clinical standard for 3D modelling of joint kinematics, implant performance and implant bearing wear is Roentgen Stereo Analysis (RSA) [5–8]. This approach involves implanting tantalum beads or markers in the bones and then taking x-rays projected through the joint in two imaging planes to measure the 3D kinematics. The reported precision (standard deviation of error) for locating the beads using this technique is 0.2° to 0.8° for rotation and 0.1 to 0.5 mm for translation [8, 9]. While the precision of this technique is considered to be acceptable, the main disadvantages of the method are a) the method can only be applied for post-operative assessment of artificial knee joints due to the requirement of implanting tantalum beads into the bone during knee replacement surgery, b) the procedure requires the patient to be exposed to ionizing radiation during the capture of the dual x-ray images. This exposure has an associated elevated risk of the patient contracting cancer and c) the x-ray images are captured while the patient is lying in a horizontal position. Hence this procedure does not allow the 3D motion analysis of the knee joint while the patient performs normal everyday activities.

Another technique is based on intra-cortical pins and bone marker [3–5]. This technique is accurate, but still highly invasive. An alternate solution for performing the required 3D motion analysis is achieved by merging 2D x-ray videos with 3D CT scans [10, 11]. It provides almost the same accuracy as RSA [12]. While this technique does not require tantalum beads to be implanted it does still have the following disadvantages: a) ionizing radiation exposure to the patient is required and b) the range of movement of the patient is limited to within the small field of view of the x-ray equipment.

The most non-invasive technique for kinematic analysis of joints is the optical tracking of skin mounted markers [13]. Commonly used products in this category are Vicon, Qualysis, Polaris, Opto-trak (Certus) and Optical Tracking Systems (Northern Digital Inc., Waterloo, ON, Canada). Although optical tracking provides sub-millimeter accuracy for kinematic analysis using skin mounted markers and is very fast for tracking multiple targets, the main disadvantage of this technique is that the markers on the skin can move independently of the underlying bone due to the flexibility of the soft muscle tissue between the bone and the skin-mounted marker. This independent movement naturally leads to soft tissue artifacts (STA).

In summary, conventional motion analysis systems utilize optoelectronic, ultrasonic or video based systems to track markers attached to the skin. These systems are non-invasive, easy-to-operate and widely used in motion analysis, computer graphics, animations and have also been proposed for computer assisted surgery [10, 14–18]. However, studies have shown that they do not provide the precision necessary which is generally 2-4 mm [19] for many clinical applications due to the large relative movement of skin and soft tissues with respect to the underlying bones during dynamic activities [20, 21]. A non-invasive ultrasound and image registration based approach will be described in the following sections to mitigate the error due to soft-tissue artifact in motion analysis of human knee joints.

## Methods

### Measuring bone position

**Figure 1:**
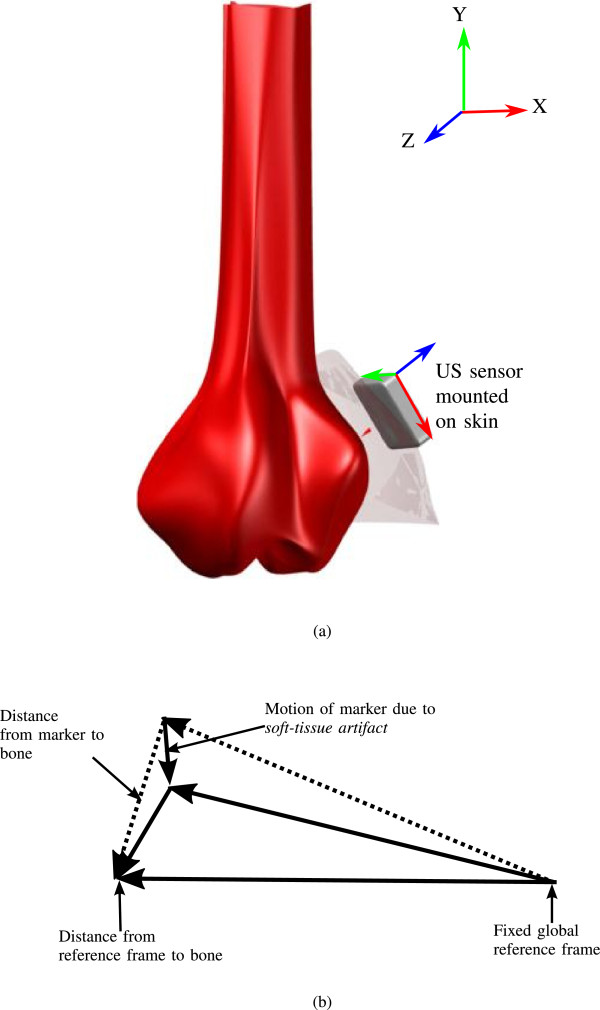
**Illustration of the proposed concepts (a) US sensor attached on skin, (b) compensating soft-tissue artifact.**

We propose to use an *intelligent* skin-mounted US sensor which contains ultrasound (US) transducers that can record images of the internal muscle tissue and bone surface while the patient is performing a particular activity. Figure 1(a) shows an illustration of this concept in 2D with the red arrow indicating the relative position of the skin-mounted sensor to the underlying bone in the knee (the femur in this case). The position of the bone relative to the skin-mounted sensor will be determined by registering the bone’s surface in an initial US frame with the bone’s surface in subsequent US frames.

Once the position of the skin-mounted sensor and the position of the bone relative to the sensor is known, these two distance vectors can be added together to find the position of the bone in a global (i.e. lab) reference frame. As shown in Figure 1(b) for the 2D case, the movement of the bone relative to the sensor can be used to compensate for the movement of the sensor due to soft-tissue artifacts. Once the soft-tissue artifact has been removed, a much more accurate measurement of the position of the bone in a global reference frame can be obtained. This measurement steps are indicated in Figure 1(b). The global reference coordinate can be established by using an optical tracker (e.g. Polaris optical tracker, Northern Digital Inc., Waterloo, ON, Canada) which usually tracks the sensor attached on the skin.

In our proposed framework, a novel *H*-shaped ultrasound array geometry is used to capture all six motion parameters for 3D rigid body movement estimation between bone and sensor. The three probes were arranged in such a way as to capture three 2D US image *slices* in a novel *H* shaped orientation. For US image acquisition, the three US arrays are mounted firmly to prevent any relative motion among the image slices as well as calibrated to ensure they preserve the *H* shape orientation. In this study, US slices are acquired with a frame rate of 30 frames per second (fps) at 24 MHz with a resolution of 0.132 mm. Five out of the six motion parameters required to describe the 3D rigid body motion can be extracted from any two orthogonal US slices but the remaining out-of-plane rotation can only be measured by using twoparallel US slices.

**Figure 2:**
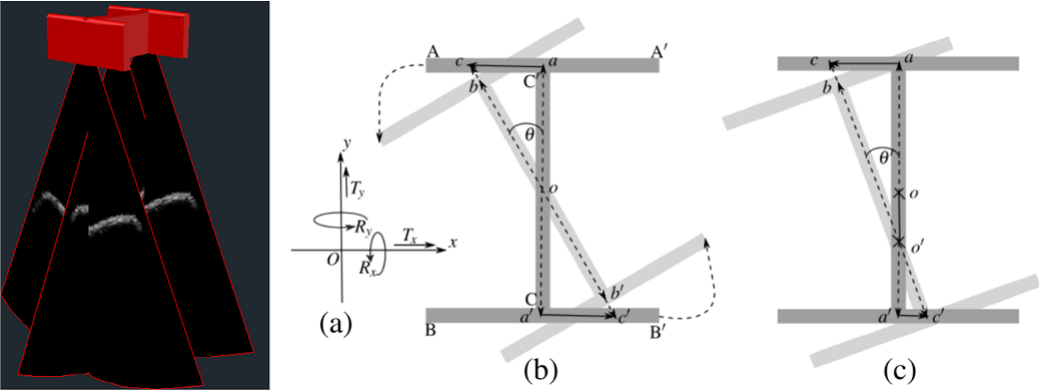
**(a) Reference sensor local coordinate system, where the** ***z*** **-axis directs into the page and not shown, (b) out-of-plane rotation (about the** ***z*** **-axis), the rotated H-shaped sensor (lighter in color) overlaid on initial position (dark) and (c) more general case, where the center of rotation,** ***o*** **shifted along slice** ***CC’*** **.**

**Figure 3:**
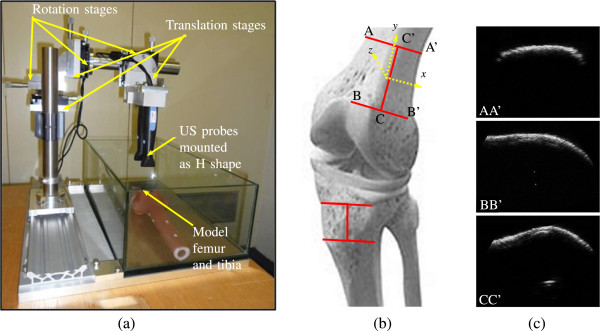
**Illustration of the first validation water-tank experiment (a) setup, (b) approximate position of US arrays, and (c) the corresponding slices.**

Three motion parameters (two translations and a rotation) can be obtained byregistering a 2D image slice to the previous one. However, to capture all six degrees-of-freedom, three slices are required. By registering these slices simultaneously to the slices from an initial US scan, the dynamic movements of the underlying bone relative to the sensor can be measured. Consider the slices *AA’*, *BB’* and *CC’* in Figures 2b and 3b. The corresponding slices from a B-mode US scan are shown in Figure 3c. The slices *AA’* and *BB’* are parallel to each other while slice *CC’* is orthogonal to both, thus forming a shape similar to the letter *H*. Slice *CC’* provides translations along the *y*- and *z*-axis respectively as well as rotation about the *x*-axis. Translation along the *x*-axis and rotation about the *y*-axis are determined by using either of the two parallel slices *AA’* or *BB’* as they are rigidly coupled and produce almost the same result. Now, for the case of out-of-plane rotation (about the *z*-axis), registering the two parallel slices (*AA’* and *BB’*) with their initial counterparts provides translations along opposite directions as shown in Figure 2b and 2c. According to the case shown in Figure 2b, the *H*-shaped slices are rotated by *θ*° about the z-axis and, *ac* and *a’c’* represent the opposite translations introduced due to this rotation. As rotation due to the soft-tissue artifacts is small (≈4.4°, [20]), the lengths *ob*≈*oc* as well as *ob*^′^≈*oc*^′^. As a result, the translations found in this case are equal and opposite, and provide a clear indication of the out-of-plane rotation about the *z*-axis. The more general case is shown in Figure 2c. In this situation, *o’c* and *o’c’* are no longer equal, however, *Δo*^′^*ac* and *Δo*^′^*a*^′^*c*^′^ are similar (according to the *law of similar triangles*), then *o*^′^*a*^′^/*aa*^′^=(*ac*+*a*^′^*c*^′^)/*a*^′^*c*^′^, and the shift of the center of rotation from *o* to *o’* can be determined. As a conclusion, we can say, however that if the two translations are equal, the axis of rotation is exactly the *z*-axis, otherwise it is parallel to the *z*-axis shifted along the slice *CC’*. It is possible, however to estimate the out-of-plane rotation using two parallel slices (*AA’* and *BB’*). Hence it turns out that the *H* shape is the most optimized geometric orientation of the US sensor for measuring all six transform parameters using low complexity 2D-2D (slice by slice) US-US image registration.

### The registration algorithm

Image registration is the process of spatially aligning one or more images to another image, generally called the reference image. It is the process of determining point to point correspondences between images of the same entity. Image registration is a versatiletechnique used in computer vision, video surveillance, automatic target recognition, medical imaging, satellite imaging and some military applications [22]. The goal of image registration in our case is to find the geometric transformation between target and reference images in order to find the relative movement between the US sensor and the underlying bone.

In the registration algorithm, a gradient descent optimization approach proposed by Lucas and Kanade [23] is used with a new similarity metric called the *sum of conditional variance* (SCV) to efficiently determine the rigid body transform parameters required to align the bone surface between two corresponding images, and this is the first article to use SCV for US-US image registration. Originally SCV was developed by our group and proposed for multi-modal medical image registration [24] but recently it has been used for mono-modal registration techniques where non-linear illumination change was an issue. In [25] the authors showed that its performance was superior when compared to the mutual information (MI) and cross-cumulative residual entropy (CCRE) similarity measures. The motivation of using SCV in our case comes from its resistance to view dependent non-linear intensity variations in US images as well as its ability to handle a large convergence radius with computational ease compared to the other popular similarity measures (e.g. sum-of-squared difference (SSD), sum-of-absolute difference (SAD) etc.).

The SCV can be calculated as follows: suppose the current US image of the bone is *I*(*x*_*i*_,*y*_*i*_) to be registered with the initial US image . The coordinates (*x*_*i*_,*y*_*i*_) and  denote the locations of pixels in *I* and *R* respectively for *i*=1…*N*, where *N* is the total number of pixels in each image. To construct the joint histogram, the images *I*_*i*_ and *R*_*i*_ were quantized from the original 256 possible values to 64 possible values. Hence, the joint histogram has 64×64 bins corresponding to the 64 possible quantized values in *I* and *R*.

Any spatial misalignment between *I* and *R* will mean that each pixel value *R*_*i*_ will correspond to a range of *I*_*i*_ values. However, typically this range of values is approximately equally distributed above and below the value for the registered version of *I*. The reason for this approximately equal distribution is explained by observing that any spatial misalignment will cause pixel values to change in opposite directions for regions of the image that exhibit opposite spatial gradients. Hence, if a spatial shift of the image causes a value of *I*_*i*_ to increase at positions with a positive spatial gradient, then this same shift will cause the same value of *I*_*i*_ to decrease in areas with negative spatial gradient and vice-versa. For the approximately equal distribution to occur, it is assumed that there is an approximately equal number of positive and negative spatial gradients in the images which is typically true for the application addressed in this paper.

The joint histogram for images *I* and *R* will be denoted by *H*(*u*,*v*) where *u* and *v* have values of 1,2,3,…*L*-1, and *L* is the number of quantized values in *I* and *R*. The values of the joint histogram represent the frequency that pixels at the same spatial location in quantized images *I* and *R* have the values *u* and *v* respectively. This joint histogram can be considered to consist of multiple conditional histograms corresponding to each of the possible quantized values of *R*_*i*_. It is possible to estimate the conditional expectation (conditional mean) of the distributions represented by these histograms using the following formula:
1

Now, given these conditional mean values, an estimate of *R*_*i*_ can be found which has pixel values that correspond linearly to the pixel values in *I*_*i*_. This estimate is found by replacing the pixel values in *R* with the conditional mean for that value of *R* and is given by:
2

for *v*=1,2,3,…,*L*-1.

The new image  now has pixel values which correspond linearly to those in *I*. However, the spatial misalignment between *I* and  will be the same as that between *I* and *R*. So the transformation required to register *I* and *R* will be the same as that required to register *I* and , but, since *I* and  are linearly related, the sum-of-the-squared difference between *I* and  can be minimized. This new similarity measure is given by:
3

Since this measure is the sum of the squared difference between values of *I*_*i*_ and their conditional mean, this equation actually represents a sum of the conditional variances (SCV) over all values of *R*_*i*_. Conveniently, since the equation for *S* now has the same form as the equation for the sum-of-squared difference, any standard optimization technique (e.g. Gauss-Newton, Levenberg Marquardt) can be used to find the geometric transform required to register  and *I*. In our experiments we minimized the SCV measure using a gradient descent optimization approach proposed by Lucas and Kanade [23].

### Experimental procedure

**Figure 4:**
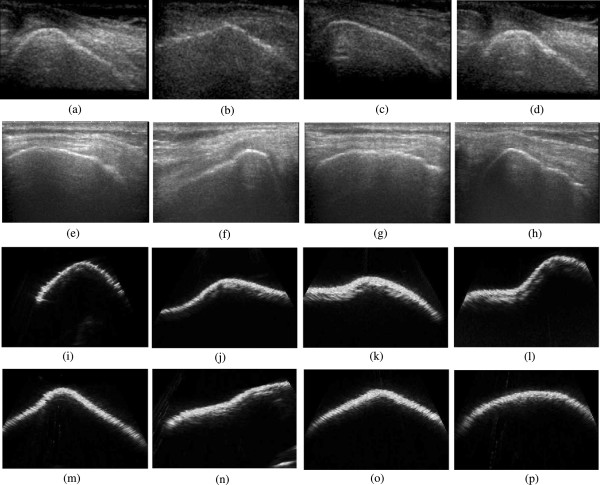
**Some** ***in-vivo*** **and** ***in-vitro*** **US scans, (a)-(d)** ***in-vivo*** **scans of the lateral femur of a human subject, (e)-(h)** ***in-vivo*** **scans of tibia, (i)-(l) US scans of a model femur, and (m)-(p) US scans of a model tibia.** The anatomical details are clearly visible in those scans, and this is crucial for non-invasive kinematic analysis.

Our kinematic analysis technique is based on the fact that knee joint provides US scans with enough anatomical details. These details (anatomical landmarks on knee bones) captured in the US scans are crucial for the accurate motion parameter estimation using image registration, which means, rotation and translation in the US scans must correspond to the similar displacements of the natural landmarks of the knee bones. Fortunately, knee joints reveal enough anatomical details to be used as natural landmarks for image registration based kinematic analysis. During the full extension of the knee joint, the posterior parts of both the distal femur and proximal tibia can be captured in US scans. However, in the 90° flexion, the anterior surfaces of the distal femur, the trochlear grove, almost all the inferior surface of the femoral condyles, the anterior superior surface of the tibia, and the anterior surface of the tibia can be easily imaged using US. Moreover, the entire medial and lateral sides of the femur and tibia are visible at all flexion angles. So, it is feasible to scan *in-vivo* the knee joints at strategic anatomical positions for the purposes of kinematic analysis. Some *in-vivo* and *in-vitro* US images are shown in Figure 4. From these images, it is apparent that the knee bones have enough details to be used for non-invasive kinematic analysis.

**Figure 5:**
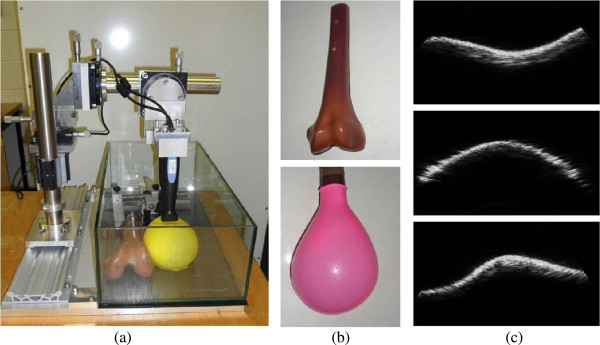
**Illustration of the second validation experiment (a) scanning setup of a model bone placed in a balloon filled with a collagen-water solution, (b) bone (top), balloon phantom (bottom), (c) typical scans using the H shaped US sensor array.**

In this validation framework for determining the movement of the sensor relative to the bones in the knee, three Interson USB ultrasound probes (Interson Corporation, Pleasanton, CA, USA) were attached to the calibration apparatus as depicted in Figure 3a and Figure 5a for two kinds of simulated environments respectively. The experimental apparatus has three rotation and three translation stages to allow the probes to be accurately positioned. The rotation stages have an angular precision of 1/60° and the translation stages have a precision of 10 *μ* m.

In the first validation experiment, an artificial model of a femur and tibia were placed in a water-filled container as shown in Figure 3a. The use of a water tank is common practice for validating ultrasound related biomedical experiments [26–28]. The water in the tank acted as a coupling medium and simulated the behaviour of muscle tissue. The frequency of the ultrasound signals used to capture the images was 24 MHz and the resolution of the images captured using these probes was 0.132 mm.

The probes were then translated by ±4 mm in steps of 0.5 mm in the *x*, *y* and *z* directions away from the initial positions and rotated by ±3° in steps of 1° around the *x*, *y* and *z* axes. At each position, the US probes were used to capture 2D B-mode scans of the surface of the model bone. These images were then registered to US images captured at the initial position of the probes. The amount of translation or rotation required to register the images was taken as an estimate of the movement of the probe relative to the surface of the bone. In-plane translations (along the *x*, *y* and *z* axes) and two in plane rotations (around the *x* and *y* axes) were determined from the two orthogonal US scans captured at the positions of *AA’* and *CC’* as well as the last out-of-plane rotation (about the *z* axis) was determined using parallel image slices at *AA’* and *BB’*, according to Figure 2. The estimates of the in-plane translations and rotations came directly from the output of the 2D-2D image registration. From the known center of rotation, the angular displacement of the two parallel probes was calculated and averaged to produce the estimate of the rotation around the *z* axis.

In the second validation experiment, we deliberately hide the internal bone structure by using an opaque balloon filled with a low concentration (10*%*, w/w at 20°C) collagen-water solution to mimic the properties of muscle tissue. This setup is shown in Figure 5. Figure 5a shows the experimental setup while Figure 5b shows the balloon phantom which covers the bone and is filled with the collagen-water solution. A typical scan from each of the three sensor arrays is also shown in Figure 5c. This kind of experimental setup can be justified by the fact that, in a real scenario we are not able to see which part of the femur and tibia are currently being scanned. As a result, there is no experimental bias introduced by placing the US probes at the best possible location for the subsequent registration process.

The low concentration collagen-water solution has been shown to closely resemble the acoustic properties of real muscle tissue [29, 30]. The speed of sound in this medium is almost 1540 m/s and collagen, the fibrous protein, is the major constituent of mammalian flesh and connective tissues [31–33]. To ensure successful transmission and reception of ultrasonic pulses through the balloon, ultrasound transmission gel was placed on the transducer and balloon interface. Then the *H* shaped US transducer assembly was moved carefully on the balloon surface to capture the translated and rotated images of the bone surface in a similar manner to the first water tank experiment. These images were then processed to extract the 6 3D rigid body transform parameters. The experiment as described in the following section, which was carried out on a human subject using accelerometer was approved by the human research ethics advisory panel (HREA) of UNSW Canberra and the approval number is A-14-31.

## Results and discussion

**Figure 6:**
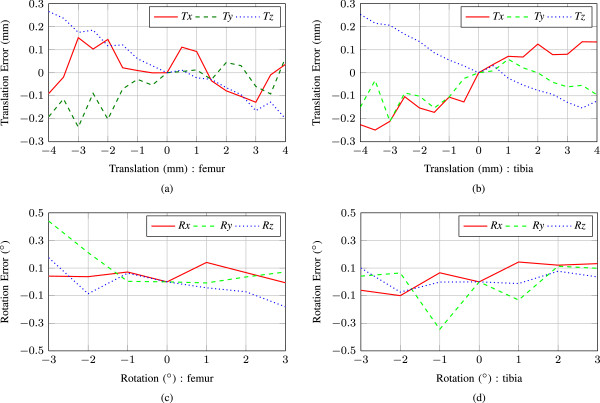
**Experiment 1 (water is used as a coupling medium between US and bone phantom): measurement errors for the proposed system.** Graphs **(a)**, **(c)** depicts errors due to femur translation and rotation estimation, and **(b)**, **(d)** for tibia translation and rotation estimation, respectively.

**Figure 7:**
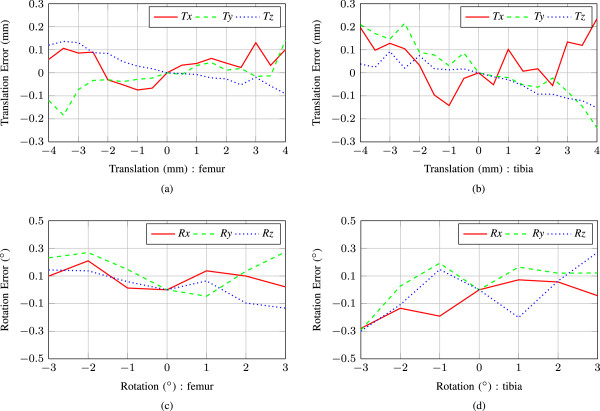
**Experiment 2 (collagen-water solution is used as a coupling medium between US and bone phantom): measurement errors for the proposed system.** Graphs **(a)**, **(c)** are for femur translation and rotation error estimation, and **(b)**, **(d)** for tibia translation and rotation, respectively.

The error between the displacements measured by the registration of the US images and the true displacements of the probes measured using the stages on the experimental apparatus are shown in Figure 6 and 7 for translation and rotation in the two simulated environments. The mean and standard deviation of these errors are shown in tabular form as well. Table 1 represents the errors introduced due to translations and rotations respectively. *Tx, Ty and Tz* denote translations in the *x, y and z* directions respectively, and *Rx, Ry and Rz* denote rotations about the *x, y and z* axes respectively. From Table 1, it is apparent that the absolute value of mean error and standard deviation range from 0.008 mm to 0.081 mm and 0.068 mm to 0.144 mm respectively for translation experiments of both tibia and femur. However, for the rotation experiments, the absolute value of mean error and standard deviation ranges are 0.010°-0.050° and 0.046°-0.160°, respectively. The relatively large mean error and standard deviation in these upper limits is due to the fact that anatomical details change slightly because of view dependent intensity variations while the probe assembly is scanning the bones in the knee joint. Also, according to Figures 6 and 7, it can be said, however, that the errors gradually increase as we move the probes further from its initial position. As the probe assembly moves away, similarity between the bone contours in corresponding slices decreases, which in turn results a larger error estimated from image registration. Commonly, the large relative errors occur at the amplitude of 3-4 mm and 2°-3°, respectively.

**Table 1:** **Mean, standard deviation (SD) of error & their averages**

			Translation (mm)				Rotation (°)			
			***Tx***	***Ty***	***Tz***	**Average**	***Rx***	***Ry***	***Rz***	**Average**
**Experiment-1**	**Femur**	**Mean**	0.009	-0.062	0.023	-0.010	0.050	0.010	-0.022	0.012
		**SD**	0.083	0.085	0.134	0.100	0.046	0.152	0.105	0.101
	**Tibia**	**Mean**	-0.028	-0.031	0.008	-0.017	0.043	-0.022	0.018	0.013
		**SD**	0.133	0.068	0.125	0.108	0.090	0.151	0.054	0.098
**Experiment-2**	**Femur**	**Mean**	-0.012	0.018	-0.013	-0.002	0.045	0.013	-0.032	0.008
		**SD**	0.092	0.092	0.144	0.109	0.056	0.156	0.112	0.108
	**Tibia**	**Mean**	0.031	-0.081	-0.011	-0.020	-0.013	-0.028	0.021	-0.006
		**SD**	0.136	0.072	0.132	0.113	0.100	0.160	0.063	0.107

**Figure 8:**
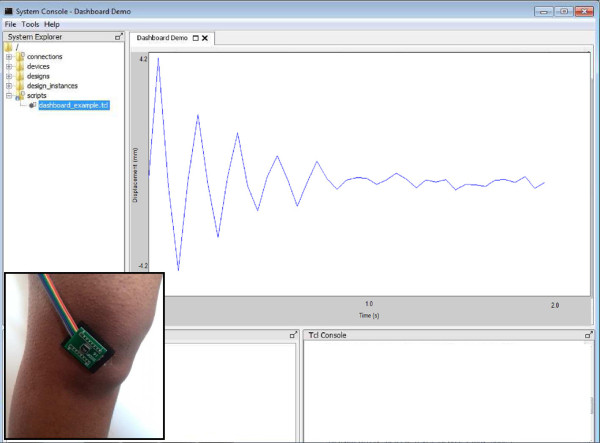
**Placement of the accelerometer on the skin [at left bottom] and data acquisition using that accelerometer after heel striking.**

In this type of motion analysis technique, two distinct frequencies are of interest- 1. The frame rate at which the position of the probe is measured in the lab reference frame and 2. The frame rate of the probes which is related to the frequency components of the relative motion between the skin and the bone. In this study, our concern was the frequency components of the relative motion of the skin and the bone using the probe assembly, which was operated at 30 Hz frame rate to capture that relative motion. To justify using 30 Hz frame rate of the US scanner used in this study, we estimated the maximum amplitude and frequency of the relative motion between skin and bone at the event of heel strike in the vertical plane by building a custom system consisting of a 3-axis digital accelerometer with 13-bit resolution (ADXL345, Analog Devices Inc, USA), which was firmly glued with the skin above the knee joint of a healthy subject (age-25, height-183 cm, weight-70 kg) as shown in Figure 8. As there was no relative motion between skin and the accelerometer, the readings from the accelerometer should reveal the necessary data, which could be used to measure the relative frequency and amplitude of motion between the bone and skin. In this experiment, the subject lifted his knee about 1/2 m, and just after striking his heel against the hard floor of the room and remained standstill, we started sampling and buffering the data at 1 ms interval, and then transferred that information to a computer (Windows-7, Intel i5 2.66 GHz) using Joint Test Action Group (JTAG) interface for post-processing. We found the amplitude of the displacement was approximately 4.2 mm, which decayed very rapidly and, it took approximately 1 s to complete about 5 cycles of vibration (thus fundamental frequency is 5 Hz) of the skin with the accelerometer. This estimation may show intra- and inter-subject variability, however, during this 1 cycle of vibration with the maximum magnitude of ±4.2 mm, the US scanner used in this study can take approximately 6 image slices, and this should be enough to capture the fundamental frequency of motion between the skin and knee bones during a sudden impact like heel strike. Moreover, from the graphs of Figures 6 and 7, it is evident that the proposed system can provide sub-millimetre accuracy when registering two US scans taken ±4 mm apart at the anatomy of interest. As a result, it is possible, however, that using 30 Hz frame rate can reliably capture motion due to soft-tissue artifact. In this study the justification using the accelerometer was performed for the event of heel strike, however, the authors believe that this type of system could be used for motion analysis during other dynamic activities (e.g. walking, jogging, running and so on). It is also reported that 500 frames per second (fps) real-time B-mode scanning is a reality nowadays, and thus the relative motion between the skin and bone during any kind of dynamic activity can be captured while incorporating our method with these advanced ultrasound scanners [34].

**Figure 9:**
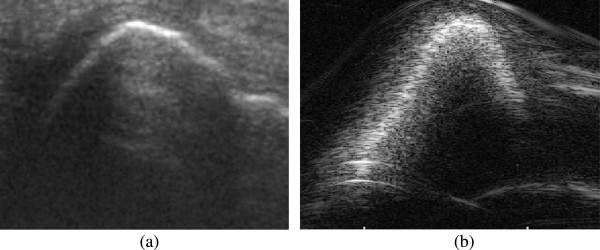
**(a) noisy in-vivo image of femoral condyle (b) corrupted in-vitro image with speckle noise (variance: 0.12).**

To quantify the effect of noise on the image registration process, we added several levels of speckle noise (Level-1: variance 0.04, Level-2: variance 0.08 and Level-3: variance 0.12) to randomly chosen image sets of tibia and femur, and performed the image registration. Speckle noise was chosen because it is the major cause of quality degradation in ultrasound images, and this form of granular noise originates from diffusive scattering and interference. Speckle noise always mask the necessary anatomical details present in US image slices and thus complicate the motion parameter estimation using image registration technique mentioned in this study. There is no specific rule to select the required levels of speckle noise necessary to be added for the validation of any image processing task and it is application specific. However, one can choose the levels of speckle noise from the proposed techniques in the literature of speckle noise reduction [35–38]. In various speckle noise reduction techniques, researchers used different levels of speckle noise such as noise variance 0.02, 0.04 or sometimes 0.08, and it is highly image and application dependent. In this study, however, we chose the noise levels which could make the in-vitro images visually similar to that of noisy in-vivo images. Figure 9(a) shows an in-vivo image of the femoral condyle and (b) shows a corrupted version of in-vitro image with speckle noise Level-3: variance 0.12. Table 2 represents the error due to various noise levels and from this table it is now evident that the proposed registration technique can be used for in-vivo images, which are usually noisier than the in-vitro images as shown in Figure 9. From Table 2, it is apparent that the absolute value of mean error and standard deviation range from 0.036 mm to 0.105 mm and 0.141 mm to 0.205 mm respectively for translation experiments of both tibia and femur. However, for the rotation experiments, the absolute value of mean error and standard deviation ranges are 0.080°-0.601° and 0.171°-0.296°, respectively. According to Table 2, mean and standard deviation of errors are lowest for the noise level 1 and highest for noise level 3.

**Table 2:** **Translation & rotation error due to different noise levels**

		Noise level-1		Noise level-2		Noise level-3	
		**Trans (mm)**	**Rot (°)**	**Trans (mm)**	**Rot (°)**	**Trans (mm)**	**Rot (°)**
**Femur**	**Mean**	0.074	-0.080	-0.089	-0.092	-0.105	0.112
	**SD**	0.141	0.211	0.169	0.243	0.200	0.296
**Tibia**	**Mean**	-0.036	0.429	-0.043	0.493	0.051	-0.601
	**SD**	0.145	0.171	0.174	0.197	0.205	0.240

In this study, the images were acquired statically at strategic anatomical positions during the experiments. However, in a dynamic activity, there may be significant amount of motion-blur induced in the acquired images, which could reduce the accuracy of this technique. Another considerable fact is the range of translation and rotation analysis performed in this study and this should be consistent with the error analysis due to the soft tissue artefact (STA), which is mainly induced by inertial effects, skin deformation, sliding and deformation caused by muscle contractions in of the knee joints. In [39], authors reported that maximum translational and rotational error occurred during dynamic activities are 11 mm and 10°, respectively, however, in this study we considered the dynamic ranges of ±4 mm and ±3°, respectively, which may require sufficiently high frame rates to capture motion, which might occur between two subsequent frames. Another issue of the proposed system may be the possible interference between two sensors attached on the knee joint to capture the relative motion between femur and tibia. However, careful selection of the anatomical positions above the joint and controlling the US beam width and directing the beam into the region of interests can virtually reduce the risk of possible interference between these sensors. In [40] the authors demonstrated a prototype for a low profile US probe with three transducer arrays each containing 96 piezoceramic elements. This probe is called *low profile* because of its small footprint and light-weight compared to the conventional US probes. Their prototype system contained integrated multiplexing electronics to reduce the size and weight of the probe and provided a viewing angle of almost 41°. This probe was specially designed for trans-rectal imaging for diagnostic assessment of prostate cancer. It is feasible however, that a similar design could be used to develop a low profile *H* shaped multi-plane US probe with embedded electronic switching circuitry which could be attached to the skin above the ends of the femur or tibia as shown in Figure 1(a). In this study, results show that the shape of the bone’s surface at the positions which were scanned by the US probe has enough detail to allow an accurate measurement of the relative position of the US probe from one scan to another, however, in some anatomical positions, bone details can be insufficient to be used in motion analysis. Also, some measurement uncertainty is introduced due to view dependent intensity variation from one US scan to another, which could further reduce the accuracy of this system. Moreover, in this method, some curvature of the bone contours is a prerequisite for successful tracking. As a result, this proposed technique can not only be used for the motion analysis of knee joints, but can be used for other human joints such as elbow, hand and hip joints provided that anatomical details in the US image slices can feasibly be captured. However, studies have shown that the necessary precision is generally 2-4 mm [19] for many clinical applications due to the large relative movement of skin and soft tissues with respect to the underlying bones during dynamic activities [20, 21]. And the precision of the proposed system compares favourably with the current clinical standard of RSA which has a reported precision of 0.2° to 0.8° for rotation and 0.1 to 0.5 mm for translation [8, 9].

## Conclusions

In this paper we have presented a novel non-invasive approach to measuring motion of the bones in a knee using tri-plane B-mode ultrasound and image registration. However, in practice, we may require to use an optical tracking system to transfer the local bone kinematic information into a global coordinate system and also build a low profileH-shaped US sensor which can be mounted on the patient’s skin using surgical tape. The experimental results reported in this paper prove that sub-millimeter precision is achievable. The use of this type of sensor would allow a system to be developed which could measure the kinematics of knee joints without restricting the patient’s movement to be confined to the field of view of imaging equipment. Such a system would provide accurate measurements while being non-invasive and would not require the use of ionising radiation.
